# Living Longer But Frailer? Temporal Trends in Life Expectancy and Frailty in Older Swedish Adults

**DOI:** 10.1093/gerona/glad212

**Published:** 2023-09-19

**Authors:** Clare Tazzeo, Debora Rizzuto, Amaia Calderón-Larrañaga, Serhiy Dekhtyar, Alberto Zucchelli, Xin Xia, Laura Fratiglioni, Davide Liborio Vetrano

**Affiliations:** Aging Research Center, Department of Neurobiology, Care Sciences and Society, Karolinska Institutet, Solna, Sweden; Aging Research Center, Department of Neurobiology, Care Sciences and Society, Karolinska Institutet, Solna, Sweden; Stockholm Gerontology Research Center, Stockholm, Sweden; Aging Research Center, Department of Neurobiology, Care Sciences and Society, Karolinska Institutet, Solna, Sweden; Stockholm Gerontology Research Center, Stockholm, Sweden; Aging Research Center, Department of Neurobiology, Care Sciences and Society, Karolinska Institutet, Solna, Sweden; Stockholm Gerontology Research Center, Stockholm, Sweden; Aging Research Center, Department of Neurobiology, Care Sciences and Society, Karolinska Institutet, Solna, Sweden; Department of Clinical and Experimental Sciences, University of Brescia, Brescia, Italy; Aging Research Center, Department of Neurobiology, Care Sciences and Society, Karolinska Institutet, Solna, Sweden; Aging Research Center, Department of Neurobiology, Care Sciences and Society, Karolinska Institutet, Solna, Sweden; Stockholm Gerontology Research Center, Stockholm, Sweden; Aging Research Center, Department of Neurobiology, Care Sciences and Society, Karolinska Institutet, Solna, Sweden; Stockholm Gerontology Research Center, Stockholm, Sweden; (Medical Sciences Section)

**Keywords:** Health trends, Longitudinal population-based study, Morbidity

## Abstract

**Background:**

This study aims to examine temporal trends in frailty state transitions, and years spent frail, in older Swedish adults.

**Methods:**

We followed the Swedish National Study on Aging and Care in Kungsholmen participants from baseline (2001–2004) for 15 (median: 9.6) years. A 40-deficit frailty index (FI) was constructed to identify 3 frailty states: robust (FI ≤ 0.125), mild frailty (0.125 < FI ≤** **0.25), and moderate and severe frailty (FI  > 0.25). Multistate survival analyses were implemented to obtain hazard ratios (HRs) for frailty state transitions, with birth year and sex as predictors. To examine temporal trends, frailty state-specific life expectancies at age 60 were forecasted for robust persons born in different years (1900, 1910, 1920, 1930, and 1940), also by sex.

**Results:**

At baseline, the 2 941 participants’ mean age was 75 years and 65% were women. Predicted life expectancy and time spent frail from age 60 followed an increasing trend by birth year. Hazards of transitioning from mild frailty to death (HR: 0.89; 95% confidence interval [CI]: 0.83–0.97) and moderate and severe frailty to death (HR: 0.98; 95% CI: 0.97–1.00) were lower for those born later. Women were less likely to transition from robust to mild frailty (HR: 0.81; 95% CI: 0.70–0.93), mild frailty to moderate and severe frailty (HR: 0.80; 95% CI: 0.68–0.93), and moderate and severe frailty to death (HR: 0.68; 95% CI: 0.59–0.78), but spent more time frail.

**Conclusions:**

Our results point to an expansion of time spent frail among older Swedish adults over time.

As in other high-income countries, life expectancy in Sweden has improved dramatically over the past century (1). This extension of life expectancy at birth has been followed by a progressive leveling off over the last decade, with the 2020 total life expectancy at birth in Sweden being 82 years (1). Now, Sweden and other countries with aging populations have the privileged but challenging task of ensuring that these years are spent in good health; an idea captured by the health-span–life-span gap, which is the difference between years lived (life span) and years lived without disease (health span) (2).

One of the greatest threats to healthy aging, frailty, is a contributor to the health span-life span gap (3). Frailty is a geriatric syndrome characterized by physiological vulnerability to stressors and inadequate functional reserves (3), leading common occurrences (eg, falls and infections) to have devastating implications. Lower quality of life and increased mortality risk for the individual, complex management for caregivers, and high health care expenditure for society are some of the reasons why frailty is burdensome (4,5). The coronavirus disease 2019 (COVID-19) pandemic has further highlighted how frailty threatens health at an individual and system level, with frail individuals infected by COVID-19 experiencing a higher risk of mortality (6).

Investigating how older adults progress through different frailty states is essential for better understanding the burden of frailty and identifying possible ways in which it can be prevented and counteracted. A systematic review on frailty state transitions, measured with frailty indices, reported that frailty state worsening is a common transition, with age being the primary driver of accelerated frailty progression (7). Low education (8), polypharmacy (9), smoking (7), and obesity (7) are some other factors that have been associated with unfavorable frailty state transitions. Frailty’s influence on healthy aging could also be assessed by examining its trends over time, which may vary due to different phenomena such as changing patterns of risk factors (ie, age, smoking, etc.) or improved diagnostics and health care tools. However, this has not yet been incorporated into research on frailty state transitions.

Two main theories on temporal trends in morbidity are (i) the compression of morbidity hypothesis, which holds that increases in life span paired with advancements in chronic disease management will compress the proportion of life lived with morbidity (10), and (ii) the expansion of morbidity hypothesis, which posits that increases in life span will be accompanied by more years lived in poor health, as the age of morbidity onset will remain the same (11). Investigations into frailty trends have led to inconsistent results, with reports of increases (12–17), reductions (18,19), and no change (20) in frailty in more recent years. Some have also examined life expectancy in different frailty states (21–26), however, to our knowledge, temporal trends of this measure have not been previously studied.

As we work to limit the dependency ratio of older to younger persons, trends in frailty progression and its influence on health span are areas that demand more attention. As such, this study aims to examine temporal trends, across years of birth, in frailty state transitions and years lived with frailty in older Swedish adults.

## Method

### Study Population

Six waves of data (between the years 2001–2004 and 2016–2019) from the Swedish National Study on Aging and Care in Kungsholmen (SNAC-K)—a population-based cohort study of older adults (community-dwelling and institutionalized) living in Stockholm, Sweden’s Kungsholmen district—were included (27). Random samples from 11 age cohorts (60, 66, 72, 78, 81, 84, 87, 90, 93, 96, and 99+ years) were invited to participate in SNAC-K, with 3 363 (73%) of eligible persons enrolled at baseline (27). Participants have been followed every 3 years if 78 years or older and every 6 years if younger than 78 (27). We included 2 941 individuals after excluding those missing frailty status at baseline (*n* = 53) and those with less than 2 observations over the 6 waves (*n* = 369; Supplementary Figure 1). Those excluded were, on average, younger, healthier, and institutionalized (Supplementary Table 1). The SNAC-K protocols were approved by the Regional Ethics Review Board in Stockholm. Participants, or proxy decision-makers for those with cognitive impairment, provided informed consent.

### Data Collection

The SNAC-K visits involved physical functioning and social interviews, clinical examinations, and neuropsychiatric assessments, by trained nurses, physicians, and psychologists, respectively (27). Where applicable, dates of death were obtained from the Swedish Cause of Death Register or from the SNAC-K records.

### Frailty Measure

We used a 40-deficit frailty index (FI), previously developed by our group, which employed a genetic algorithm to select the number and nature of deficits to generate the best predictive FI (area under the curve for 3-year mortality = 0.88; 95% CI = 0.85–0.91) for the baseline SNAC-K population (28). There were 40 deficits (Supplementary Table 2) included: 19 chronic diseases assessed by physicians through information from clinical examinations, participant-reported health, lab parameters, medication utilization, and medical records (29); 13 records of physical performance/function assessed during the physician and nurse examinations; 4 socioeconomic factors obtained by nurses through the social interview; one sign (abnormal patellar reflex) measured in the clinical examination; one health problem (loss of appetite) ascertained from the physician interview; one cognition measure (Mini Mental State Examination) obtained during the neuropsychiatric assessment; and one health care utilization measure (≥1 acute hospitalizations in the past year) obtained from the Swedish National Patient Register and physician interview.

Frailty measures were ascertained each time a participant attended a study visit during the first 6 waves of SNAC-K. The FI score was computed by dividing the number of deficits that a participant displayed by the total number of nonmissing deficits considered. In cases where the missing deficit count exceeded 4 (10% of deficits), the FI score was set to missing ( Supplementary Table 3). We then generated frailty states to increase the clinical and public health interpretability of our results. To generate frailty state categories, we employed the method introduced by Clegg et al. (30), where the 99th centile of baseline FI scores for the study population was selected as the upper limit and 4 categories of equal FI score distance were created. To facilitate model convergence, the upper 2 quartiles were combined, resulting in 3 frailty state categories: robust (FI ≤ 0.125), mild frailty (0.125 < FI ≤** **0.25), and moderate and severe frailty (FI > 0.25).

### Statistical Analysis

Descriptive baseline statistics of the participants were analyzed by frailty state through chi-square tests and one-way analyses of variance. The movement of participants across different frailty states, death, loss-to-follow-up, and missing data were presented as an alluvial plot. Multistate survival analyses were carried out to describe the movement of participants between different frailty states and death, relying on the Markov assumption that one’s future state is only informed by their current, and not past, state (31). Using the multistate models, frailty state transitions were examined at 5 transition times (between the 6 waves of SNAC-K). These transitions occurred between 3 transient states corresponding to different degrees of frailty status (robust, mild frailty, and moderate and severe frailty) and death as an absorbing state (Supplementary Figure 2). Birth year was operationalized as a continuous variable. Hazard ratios (HRs) and 95% confidence intervals (CIs) for the different frailty state transitions were obtained with birth year and sex as predictors; age was included as a time-varying covariate. Based on the maximum likelihood estimates of the multistate analyses, life expectancies at age 60 in different frailty states were predicted for hypothetical robust persons born in five different years (1900, 1910, 1920, 1930, and 1940), also stratified by sex. Simulations were carried out to estimate the uncertainty, allowing for 95% CIs of the life expectancy estimates to be presented. Using our data from 2001 to 2019, we estimated life expectancy at the same age (60 years), an established health indicator (32), for robust persons born in different years to assess whether there has been a compression or expansion of frailty. The life expectancy estimates were presented as an absolute number of years, as well proportion of time, from age 60 spent in the different frailty states. Statistical significance was set as a *p* value <.05. All analyses were conducted using Stata/SE 17.0 (StataCorp LLC, College Station, TX) and R version 4.2.1. Please see the Appendix in the Supplement for additional details on the statistical analyses and R packages.

### Sensitivity Analyses

Two sensitivity analyses were carried out. First, FIs are traditionally comprised of physiological deficits to reflect biological aging processes (33). Our FI contains socioeconomic and care-related factors that deviate from this traditional idea of a FI; as such, the multistate survival analyses were re-run with a modified FI that removed any nonphysiological deficits (Supplementary Table 2). Second, dropping out was included as an additional state in the multistate survival model to assess how dropouts might have influenced the primary analyses.

## Results

**Figure 1.**
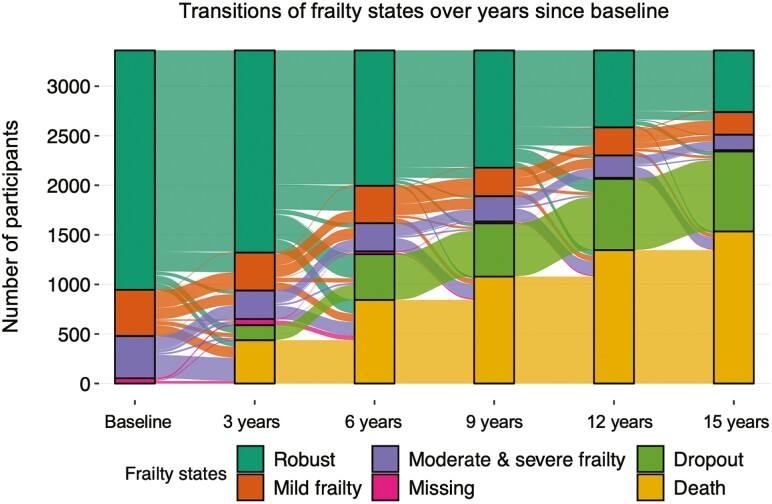
Alluvial plot with transitions in frailty states over 15 years of follow-up.

At baseline, the mean age of the 2 941 participants was 75 years and 65% were women. The baseline characteristics of the participants by frailty status can be found in Table 1. Across the 5 transition times, participants most frequently maintained their frailty status, transitioned to a more severe frailty status, died, or dropped out of the study (Figure 1; Supplementary Tables 4–5). Those born in later birth years had a statistically significant reduced hazard (per 1-year increase in birth year) for transitioning from mild frailty to death (HR: 0.89; 95% CI: 0.83–0.97) and from moderate and severe frailty to death (HR: 0.98; 95% CI: 0.97–1.00; Table 2). In comparison to men, women had lower hazards for transitioning from robust to mild frailty (HR: 0.81; 95% CI: 0.70–0.93), from mild frailty to moderate and severe frailty (HR: 0.80; 95% CI: 0.68–0.93), and from moderate and severe frailty to death (HR: 0.68; 95% CI: 0.59–0.78; Table 2).

**Table 1.** Participant Characteristics by Frailty State at Baseline

Characteristics	Robust (*n* = 2 122; 72.2%)	Mild Frailty (*n* = 414; 14.1%)	Moderate and Severe Frailty (*n* = 405; 13.8%)	Total (*n* = 2 941, 100.0%)
Age	70.4 ± 8.8	83.7 ± 7.7	89.3 ± 7.0	74.8 ± 11.2^*^
Sex (women)	1279 (60.3)	296 (71.5)	327 (80.7)	1902 (64.7)^*^
Birth year	1932.1 ± 9.0	1918.9 ± 7.7	1913.3 ± 7.1	1927.6 ± 11.3^*^
Elementary education	210 (9.9)	126 (30.5)	175 (44.0)	511 (17.4)^*^
Walking speed < 0.8 m/s	199 (9.5)	312 (77.2)	329 (96.5)	840 (29.5)^*^
MMSE score < 27	74 (3.5)	129 (31.2)	317 (79.7)	520 (17.8)^*^
Institutionalized	1 (0.1)	7 (1.7)	152 (37.5)	160 (5.4)^*^
1+ impaired ADLs	13 (0.6)	32 (7.8)	228 (56.7)	273 (9.3)^*^
1+ impaired IADLs	77 (3.7)	212 (54.2)	338 (98.8)	627 (22.3)^*^
Chronic diseases				
Heart failure	66 (3.1)	101 (24.4)	156 (38.5)	323 (11.0)^*^
Ischemic heart disease	222 (10.5)	112 (27.1)	123 (30.4)	457 (15.5)^*^
Atrial fibrillation	108 (5.1)	84 (20.3)	94 (23.2)	286 (9.7)^*^
Dementia	9 (0.4)	40 (9.7)	227 (56.1)	276 (9.4)^*^
Depression	147 (6.9)	48 (11.6)	70 (17.3)	265 (9.0)^*^
Cerebrovascular disease	69 (3.3)	64 (15.5)	100 (24.7)	233 (7.9)^*^
COPD	64 (3.0)	47 (11.4)	43 (10.6)	154 (5.2)^*^
Cancer	170 (8.0)	58 (14.0)	48 (11.9)	276 (9.4)^*^
Chronic kidney disease	555 (26.2)	246 (59.4)	203 (50.1)	1004 (34.1)^*^
Anemia	111 (5.2)	105 (25.4)	145 (35.8)	361 (12.3)^*^
Number of chronic diseases	3.3 ± 1.9	5.8 ± 2.4	6.7 ± 2.8	4.1 ± 2.5^*^

*Notes.* MMSE = Mini Mental State Examination; 1 + impaired ADLs = one or more impaired activities of daily living; 1 + impaired IADLs = one or more impaired instrumental activities of daily living; COPD = chronic obstructive pulmonary disease. Missing variables: elementary education (*n* = 8); walking speed (*n* = 92); MMSE (*n* = 12); ADLs (*n* = 6); and IADLs (*n* = 130). Values are presented as absolute number and column percentage (%) or mean ± standard deviation.

^*^ *p* < .001.

**Table 2.** Hazard Ratios and 95% Confidence Intervals for Transitions Between Frailty States With Birth Year and Sex as Predictors

Transitions			Birth Year (per 1 year increase)	Sex (women vs men)
From	To	***N***	HR (95% CI)	HR (95% CI)
Robust	Mild frailty	832	0.99 (0.98–1.01)	**0.81 (0.70–0.93)**
Robust	Death	313	1.00 (0.89–1.12)	0.50 (0.24–1.07)
Mild frailty	Robust	42	1.06 (0.98–1.15)	0.82 (0.43–1.57)
Mild frailty	Moderate and severe frailty	498	0.99 (0.97–1.01)	**0.80 (0.68–0.93)**
Mild frailty	Death	346	**0.89 (0.83–0.97)**	0.69 (0.45–1.05)
Moderate and severe frailty	Mild frailty	14	0.90 (0.78–1.04)	0.89 (0.28–2.81)
Moderate and severe frailty	Death	753	**0.98 (0.97–1.00)**	**0.68 (0.59–0.78)**

*Notes:* CI = confidence interval; HR = hazard ratio. The model for birth year is adjusted by sex (women) and age (time-varying). The model for sex (women) is adjusted by birth year and age (time-varying). HRs in bold are statistically significant (*p* value <.05).

**Figure 2.**
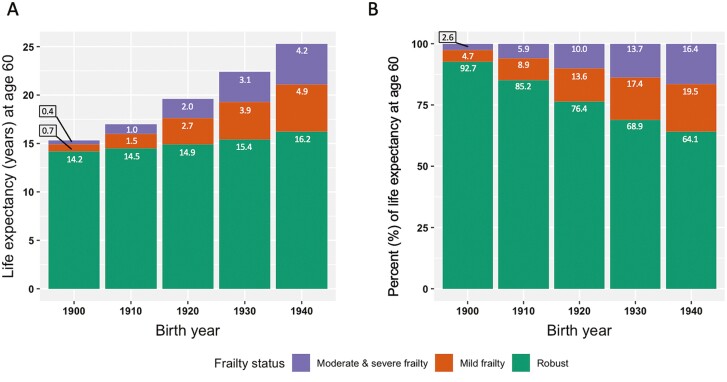
Forecasted life expectancy in different states of frailty for robust persons aged 60 and across birth years, in absolute numbers (**A**) and as percentage of total remaining life (**B**).

**Figure 3.**
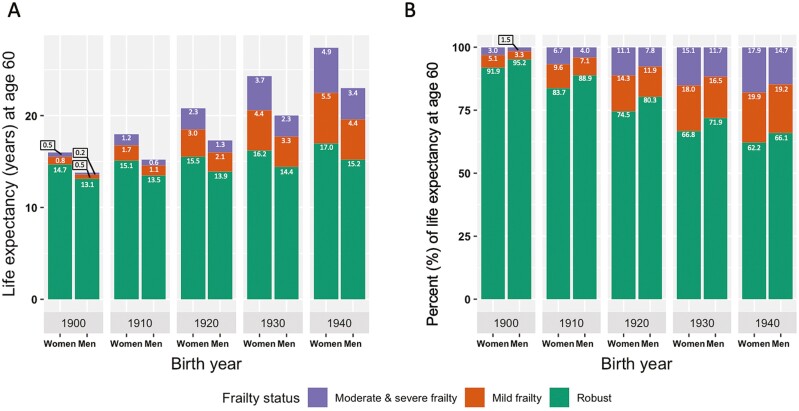
Forecasted life expectancy in different states of frailty for robust persons aged 60 and across birth years and by sex, in absolute numbers (**A**) and as percentage of total remaining life (**B**).

Forecasted life expectancy at age 60 for robust persons increased over time, with 15.3 years for those born in 1900, and 25.3 years for those born in 1940 (Figure 2). Our estimates were not statistically significant, however, clear trends emerged, suggesting that more life years were spent in each frailty state and that the proportion of life spent frail from age 60 increased in more recent birth years (Figure 2). The predicted proportion of life from age 60 spent in the states of mild, or moderate and severe, frailty increased from 7.3% for those born in 1900, to 35.9% for those born in 1940. Although not statistically significant, our estimates suggest that women had a greater life expectancy at age 60 than men across all five birth years (Figure 3). For those born in 1900, men’s total forecasted life expectancy at age 60 was lower than women’s forecasted robust life expectancy at age 60, but for those born in 1940, men’s total forecasted life expectancy at age 60 exceeded women’s forecasted life expectancy at age 60 in the robust and mild frailty states (Figure 3). Compared to women, our results suggest that men spent a lower proportion of their life remaining at age 60 frail across all five birth years (Figure 3). The 95% CIs for the predicted life expectancies across all birth years in the study population, also by sex, are presented in Supplementary Figure 3. The life expectancy estimates were more precise for those born between 1920 and 1940, and less precise in other birth years, due to limited data.

In terms of the sensitivity analyses, the HRs and 95% CIs for the transitions between frailty states, using a modified FI that excluded nonphysiological deficits, show similar results to the main analyses (Supplementary Table 6). The only notable difference is that being born later became statistically significantly protective against the transition from mild frailty to moderate and severe frailty (HR: 0.97; 95% CI: 0.95–0.99), as well as from moderate and severe to mild frailty (HR: 0.78; 95% CI: 0.63–0.97); the results of the main analyses are in the same direction, but just short of being statistically significant. The sensitivity analysis that included dropping out as an additional state showed very similar results to the main analyses, with the transitions being statistically significant in the same direction and with similar magnitude (Supplementary Table 7).

## Discussion

Our study led to three relevant findings: first, the likelihood of transitioning toward frailty remained similar for those born in later years, but frailty was less deadly; second, although not statistically significant, our life expectancy estimates suggest an expansion of frailty in later-born older Swedish adults; and third, women lived longer, but with more time spent frail, than men.

Our results suggest an expansion of life expectancy and frailty from age 60 in older adults, as well as a progressive delay in the onset of frailty, for those born in later years. We observed a greater proportion of life spent frail, or in other words a greater health-span–life-span gap, after age 60 for those born more recently. However, frailty was also less deadly for these later-born individuals. Two potential explanations for this expansion of, less deadly, frailty are that the deficits have become less severe and easier to control medically and that there has been a change in the collection of deficits driving frailty over the years. Individuals born later can arguably live longer with FI deficits because of improved care for previously deadly acute conditions (eg, stroke, myocardial infarction, cancer), and increased secondary and tertiary prevention strategies, which make living with frailty more manageable (34). Another Swedish study found frailty to be less associated with mortality in later-born cohorts (20), also supporting the notion that later cohorts can live longer with frailty.

We can speculate about the reason behind these life expectancy trends by contextualizing our results in terms of major events of the past century. The early 20th century included crises (eg, World Wars I and II, the 1918 Influenza Pandemic, and Great Depression) and scientific and societal advancements (eg, antibiotics, vaccinations, compulsory education, increased income per capita, and welfare) that could have influenced health and life expectancy (35). Older generations experienced many crises, and these crises occurred during the potentially critical or sensitive periods of childhood and adolescence, that could have resulted in lasting physiological consequences (36). Those born more recently were also exposed to negative events (eg, World War II); however, by this time, health care had improved, possibly lessening the shock of such events (35). As such, one could say that those born in more recent years had a relative advantage; they reaped the benefits of advancements and avoided the negative implications of crises to a greater degree, enabling them to live longer but at the expense of becoming frail (11). Moreover, survival of the fittest might be relevant, with those born earlier who survived the hardships of the earlier twentieth century representing a more robust group.

Our findings on life expectancy by sex are consistent with the well-established male–female health-survival paradox: men are more likely to die across the life course, but women live in worse health (37). More recently, the sex-frailty paradox—women are frailer but less susceptible to death—was conceptualized (38). Our results are in line with this paradox, as women had a longer predicted life expectancy at age 60 across all birth years, with a greater proportion of time spent mildly, and moderately and severely, frail. Interestingly, women had lower hazards of several unfavorable frailty state transitions, which is likely because they can bear frailty longer than men, making them less likely to transition to worse states. In other words, women have more stable health statuses, and are less likely to die, but they spend more time frail and accrue more deficits. This phenomenon of longer life expectancy for women, but also longer time with frailty, has been reported by others as well (21–26). Differences in health care seeking, risky behavior, hormonal and immunological factors, and the stressful nature of women’s societal roles are among many potential explanations for this paradox (37,38). It is also possible that certain deficits have differential consequences and frequencies depending on sex (eg, being widowed, cardiovascular disease), and because the FI does not weigh deficits differently, this might mean differential frailty severity for men and women.

Our life expectancy forecasts also show a higher concordance between the sexes in more recent birth years concerning time spent in the different frailty states. This finding can also be contextualized in terms of historical events of the 20th century. Societal roles became less segregated by sex in Sweden; more Swedish women participated in the workforce in the last half of the twentieth century (39) and gender equality policies (eg, parental leave for both men and women, subsidized childcare, and flexible working conditions) (40) have allowed men and women to be exposed to the associated benefits and consequences of their environments to a similar degree. Moreover, the changing demographic of smoking and associated behavior (eg, alcohol consumption, reduced physical activity), with a rise in women and a reduction in men (41) helps to explain this increased overlap in frailty state-specific life expectancy by sex.

This study has several limitations. First, we only allowed transitions between adjacent frailty states to facilitate model convergence. However, it is theoretically possible—albeit unlikely—for a person to skip states from robust to moderate and severe frailty in an instant. Second, the SNAC-K panel data structure only allows frailty to be measured every 3–6 years, but it is possible that state changes could have occurred between study waves; interval censoring was used to account for this. Third, the results might be unfair to those born more recently, as improvements in life expectancy might be underestimated due to survivorship bias in those born earlier. Fourth, loss-to-follow-up might have influenced the study results; however, in the sensitivity analysis, dropping out was included as an additional state, with very similar results to the main analyses. Fifth, in our FI, once a person was diagnosed with a chronic disease, they were said to have that deficit for all future waves; it is possible that some participants might not have been affected by certain chronic diseases anymore, which might have prevented us from detecting backward transitions. However, our FI was not limited to chronic diseases, also containing care and physical function measures as well as socioeconomic factors that would have allowed for the detection of backward transitions. Sixth, it was not possible to follow all participants from age 60 to death; thus, the life expectancy estimates are predictions based on the fitted multistate model. Finally, caution should be paid to the results’ generalization, as the SNAC-K participants are relatively affluent and primarily white. The frailty prevalence is in line with the literature (42), however, life expectancy predictions are likely inflated due to the relative advantage of Kungsholmen residents, who, on average, have some of the highest total life expectancies, self-reported health, average incomes, and educational attainments of all the Stockholm municipalities (43). Socioeconomic factors have been tied to inequalities in life expectancy (44); it is likely that the privilege held by residents of Kungsholmen translates into longer forecasted life expectancies at age 60 compared to Stockholm and Sweden.

As population aging continues, it is more important than ever that we monitor trends in frailty (3,5). Future research should identify temporal trends in the deficits that are driving the frailty burden as well as modifiable risk factors that can be addressed to delay and reverse frailty progression. Along these lines, research on time spent frail across socioeconomic indicators should be conducted so we can learn about and prevent inequities in healthy life expectancy. It is important that we raise our expectations for what constitutes acceptable health in older age and aim for a society where older adults can be active, thriving parts of their communities. Reducing the health-span–life-span gap will not only have positive implications for older adults but also for society and the health care system.

In conclusion, the results of this study point to a bittersweet message: that older adults in Sweden are living longer, but at the price of more time spent frail. Our results suggest there might have been an expansion of frailty in older Swedish adults. In addition, we found unfavorable transitions to frailty and death are less likely to occur in women and those born in later years. Continuing to improve our understanding of frailty trends will be essential for resource allocation, care design, and preventing a dependency among older adults that cannot be sustained by the smaller, younger generation.

## Supplementary Material

glad212_suppl_Supplementary_Material

## Data Availability

Data are from the SNAC-K project, a population-based study on aging and dementia (http://www.snac-k.se/). Access to these original data is available to the research community upon approval by the SNAC-K data management and maintenance committee. Applications for accessing these data can be submitted to Maria Wahlberg (Maria.Wahlberg@ki.se) at the Aging Research Center, Karolinska Institutet.
