# Supplementary material for: Living Longer But Frailer? Temporal Trends in Life Expectancy and Frailty in Older Swedish Adults
Source: J Gerontol A Biol Sci Med Sci. 2023 Sep 19;79(1):glad212. doi: 10.1093/gerona/glad212 (PMC10733192; doi:10.1093/gerona/glad212)
Supplement: glad212_suppl_Supplementary_Material [file glad212_suppl_supplementary_material.pdf]

Supplemental Material

Page 2 - eFigure 1: Flowchart of study participation

Page 3 - eFigure 2: Directed graph of permitted transitions in the multi-state survival model

Page 4 - eFigure 3: Forecasted frailty state-specific life expectancy at age 60 with 95% confidence intervals by birth year in the total study population (A), women (B), and men (C)

Page 5 - eTable 1: Characteristics of included versus excluded participants at baseline

Page 6 - eTable 2: 40 deficits included in the frailty index

Page 7 - eTable 3: Participants with missing frailty status (missing more than 10% of deficits)

Page 8 - eTable 4: Number of participants in different frailty states and deaths across study waves

Page 9 - eTable 5: Overall number of transitions between frailty states and death

Page 10 - eTable 6: Hazard ratios and 95% confidence intervals for transitions between frailty states using a modified frailty index

Page 11 - eTable 7: Hazard ratios and 95% confidence intervals for transitions between frailty states with dropouts as an additional state

Page 12 - eTable 8: Distribution of participants by birth year

Page 13 - Appendix

Page 14 - References

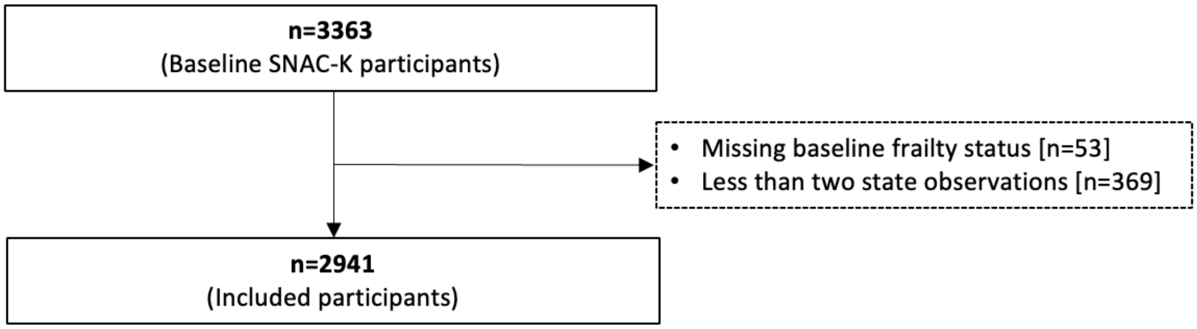

eFigure 1: Flowchart of study participation

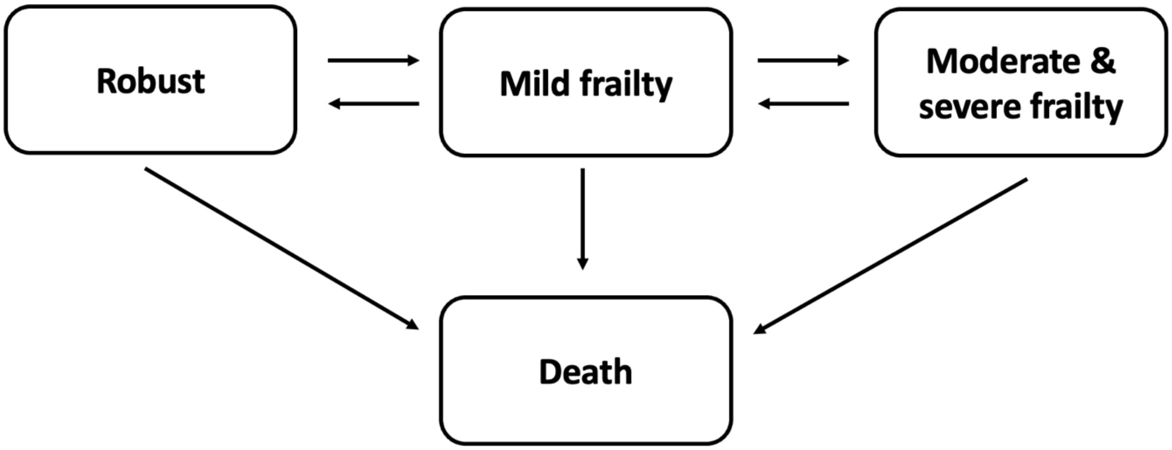

eFigure 2: Directed graph of permitted transitions in the multi-state survival model

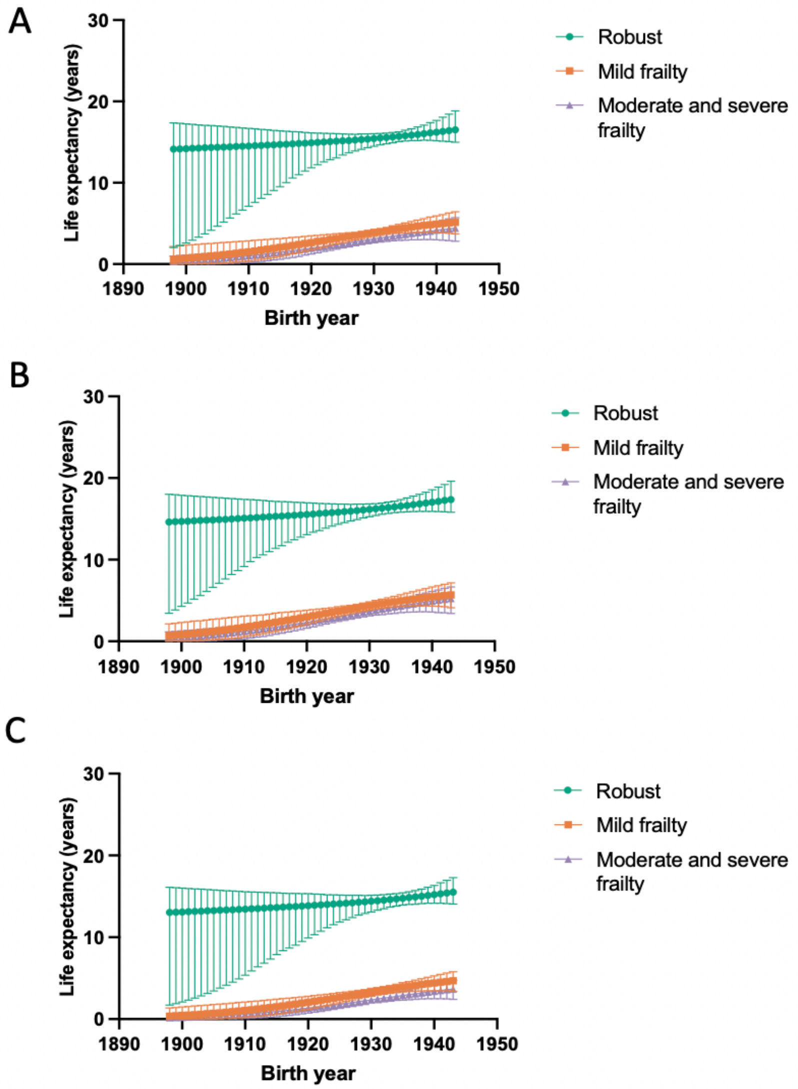

eFigure 3: Forecasted frailty state-specific life expectancy at age 60 with 95% confidence intervals by birth year in the total study population (A), women (B), and men (C)

eTable 1: Characteristics of included versus excluded participants at baseline

Characteristics	Included (n=2941; 87.4%)	Excluded (n=422; 12.6%)	Total (n=3363, 100.0%)
**Age**	74.8 ± 11.2	73.8 ± 11.1	74.7 ± 11.2
**Sex (women)**	1902 (64.7)	280 (66.4)	2182 (64.9)
**Elementary education**	511 (17.4)	79 (19.9)	590 (17.7)
**Walking speed < 0.8m/s**	840 (29.5)	130 (33.2)	970 (29.9)
**MMSE score < 27**	520 (17.8)	82 (20.2)	602 (18.1)
**Institutionalized**	160 (5.4)	35 (8.3)	195 (5.8)*
**1+ impaired ADLs**	273 (9.3)	49 (12.0)	322 (9.6)
**1+ impaired IADLs**	627 (22.3)	83 (22.0)	710 (22.3)
**Chronic diseases**			
**Heart failure**	323 (11.0)	30 (7.1)	353 (10.5)*
**Ischemic heart disease**	457 (15.5)	57 (13.5)	514 (15.3)
**Atrial fibrillation**	286 (9.7)	38 (9.0)	324 (9.6)
**Dementia**	276 (9.4)	46 (10.9)	322 (9.6)
**Depression**	265 (9.0)	45 (10.7)	310 (9.2)
**Cerebrovascular disease**	233 (7.9)	32 (7.6)	265 (7.9)
**COPD**	154 (5.2)	13 (3.1)	167 (5.0)
**Cancer**	276 (9.4)	23 (5.5)	299 (8.9)*
**Chronic kidney disease**	1004 (34.1)	113 (26.8)	1117 (33.2)*
**Anemia**	361 (12.3)	44 (10.4)	405 (12.0)
**Number of chronic diseases**	4.1 ± 2.5	3.8 ± 2.3	4.1 ± 2.5*

Notes: MMSE = Mini Mental State Examination; ADLs = Activities of daily living; IADLs = Instrumental activities of daily living; COPD = Chronic obstructive pulmonary disease. Missing variables: elementary education ($n = 32$); walking speed < 0.8m/s ($n = 122$); MMSE < 27 ($n = 27$); 1+ impaired ADLs ($n = 19$); 1+ impaired IADLs ($n = 174$). Values are presented as absolute number and column percentage (%) or mean ± standard deviation.

[*] $P < 0.05$.

eTable 2: 40 deficits included in the frailty index

Domain	Deficit
**Chronic Diseases[a]**	Anaemia
	Deafness and other hearing loss
	Heart failure
	Atrial fibrillation
	Dementia
	Solid neoplasms
	Cerebrovascular diseases
	Chronic obstructive pulmonary disease
	Other eye diseases
	Blindness and other visual loss problems
	Other cardiovascular disorder
	Cardiac valve disorders
	Other psychiatric disorders
	Other neurological disorders
	Bradycardias and other cardiac conduction disorders
	Peripheral vascular disease
	Chronic diseases of pancreas and gallbladder
	Parkinson's disease and parkinsonism
	Other respiratory disorders
**Physical performance/function measures**	Physical inactivity (engaging in physical activity 2-3 times/month or less)
	Walking speed < 0.8 metres/second
	Using a walking aid
	Inability to do laundry alone
	Inability to prepare and take medicines alone
	Inability to prepare food alone
	Inability to use means of transportation alone
	Inability to manage finances alone
	Inability to wash him/herself alone
	Inability to use the toilet alone
	Inability to dress alone
	Inability to feed him/herself alone
	Inability to transfer from bed to chair alone
**Socioeconomic factors**	Poor social network*
	Being widowed*
	Elementary education*
	Living in a nursing/retirement home*
**Sign**	Abnormal patellar reflex
**Health problem**	Reporting loss of appetite
**Cognition measure**	Mini Mental State Examination score < 27
**Healthcare utilization measure**	1+ acute hospitalization in previous year*

* The asterisk denotes the 5 non-physiological deficits excluded from the 35-deficit modified frailty index.

[a] The chronic diseases were measured at the time of the study visit. If an individual was diagnosed with a chronic disease in one wave, they were considered to have it in all following waves.

eTable 3: Participants with missing frailty status (missing more than 10% of deficits)

Study wave	Number of participants	Number and percent [n (%)] of participants with missing frailty status
**1 (baseline)**	3363	53 (1.6)
**2 (3-year follow-up)**	990	52 (5.3)
**3 (6-year follow-up)**	2058	28 (1.4)
**4 (9-year follow-up)**	702	18 (2.6)
**5 (12-year follow-up)**	1298	10 (0.8)
**6 (15-year follow-up)**	518	14 (2.7)

eTable 4: Number of participants in different frailty states and deaths across study waves

Wave	Robust	Mild frailty	Moderate and severe frailty	Death
**1 (baseline)**	2122	414	405	N/A
**2 (3-year follow-up)**	350	320	265	411
**3 (6-year follow-up)**	1367	377	283	397
**4 (9-year follow-up)**	214	225	245	232
**5 (12-year follow-up)**	778	284	226	266
**6 (15-year follow-up)**	191	171	142	189

eTable 5: Overall number of transitions between frailty states and death

Transition	Number of transitions
Robust → Robust	2857
Robust → Mild frailty	832
Robust → Moderate and severe frailty	145
Robust → Death	313
Mild frailty → Robust	42
Mild frailty → Mild frailty	531
Mild frailty → Moderate and severe frailty	498
Mild frailty → Death	346
Moderate and severe frailty → Robust	1
Moderate and severe frailty → Mild frailty	14
Moderate and severe frailty → Moderate and severe frailty	507
Moderate and severe frailty → Death	753

eTable 6: Hazard ratios and 95% confidence intervals for transitions between frailty states using a modified frailty index

**Transitions**		**Birth year (per one year increase)**	**Sex (women)**
**From**	**To**	**HR (95% CI)**	**HR (95% CI)**
**Robust**	**Mild frailty**	1.00 (0.98-1.02)	**0.74 (0.65-0.86)**
**Robust**	**Death**	0.98 (0.87-1.09)	0.58 (0.29-1.15)
**Mild frailty**	**Robust**	1.01 (0.91-1.11)	1.07 (0.46-2.48)
**Mild frailty**	**Moderate & severe frailty**	**0.97 (0.95-0.99)**	**0.83 (0.71-0.97)**
**Mild frailty**	**Death**	**0.90 (0.82-0.98)**	0.71 (0.44-1.15)
**Moderate & severe frailty**	**Mild frailty**	**0.78 (0.63-0.97)**	1.09 (0.22-5.32)
**Moderate & severe frailty**	**Death**	**0.98 (0.96-0.99)**	**0.71 (0.62-0.81)**

Note: HR = hazard ratio, CI = confidence interval. The modified frailty index has 35 deficits. Five non-physiological deficits were dropped: poor social network, being widowed, elementary education, at least one acute hospitalization in the previous year, and living in a nursing/retirement home. A FI score was set to missing if the participant was missing more than 3 deficits (>8.6% missing). The model for birth year is adjusted by sex (female) and age (time-varying). The model for sex (female) is adjusted by birth year and age (time-varying). HRs in bold are statistically significant (p-value < 0.05).

eTable 7: Hazard ratios and 95% confidence intervals for transitions between frailty states with dropouts as an additional state

**Transitions**		**Birth year (per one year increase)**	**Sex (women)**
**From**	**To**	**HR (95% CI)**	**HR (95% CI)**
**Robust**	**Mild frailty**	0.99 (0.97-1.01)	**0.84 (0.73-0.97)**
**Robust**	**Dropout**	1.03 (0.99-1.08)	0.89 (0.69-1.15)
**Robust**	**Death**	1.00 (0.89-1.11)	0.50 (0.23-1.06)
**Mild frailty**	**Robust**	1.06 (0.98-1.14)	0.77 (0.40-1.48)
**Mild frailty**	**Moderate & severe frailty**	0.99 (0.97-1.01)	**0.77 (0.66-0.90)**
**Mild frailty**	**Dropout**	0.99 (0.94-1.05)	1.63 (0.95-2.80)
**Mild frailty**	**Death**	**0.90 (0.83-0.97)**	0.69 (0.44-1.07)
**Moderate & severe frailty**	**Mild frailty**	0.90 (0.78-1.04)	0.96 (0.30-3.14)
**Moderate & severe frailty**	**Dropout**	1.02 (0.97-1.08)	0.92 (0.57-1.50)
**Moderate & severe frailty**	**Death**	**0.98 (0.96-0.99)**	**0.67 (0.58-0.77)**

Notes: HR = hazard ratio, CI = confidence interval. The model for birth year is adjusted by sex (women) and age (time-varying). The model for sex (women) is adjusted by birth year and age (time-varying). HRs in bold are statistically significant (p-value < 0.05).

eTable 8: Distribution of participants by birth year

Birth year	Number and percent [n (%)] of participants
1898	1 (0.03)
1899	3 (0.10)
1900	3 (0.10)
1901	6 (0.20)
1902	5 (0.17)
1903	5 (0.17)
1904	5 (0.17)
1905	15 (0.51)
1906	19 (0.65)
1907	27 (0.92)
1908	45 (1.53)
1909	22 (0.75)
1910	56 (1.90)
1911	90 (3.06)
1912	66 (2.24)
1913	67 (2.28)
1914	43 (1.46)
1915	34 (1.16)
1916	74 (2.52)
1917	55 (1.87)
1918	37 (1.26)
1919	106 (3.60)
1920	85 (2.89)
1921	48 (1.63)
1922	70 (2.38)
1923	137 (4.66)
1924	85 (2.89)
1925	186 (6.32)
1929	131 (4.45)
1930	95 (3.23)
1931	200 (6.80)
1935	145 (4.93)
1936	127 (4.32)
1937	213 (7.24)
1941	213 (7.24)
1942	149 (5.07)
1943	273 (9.28)

Appendix

R packages

The following R packages were employed in the analyses: 1) ggalluvial for the alluvial plot (1), 2) msm for the multi-state survival analyses (2), and 3) ELECT for the state-specific life expectancies (3).

Multi-state models

Multi-state survival models were employed in this study. Such models operate under the Markov assumption that transition probabilities and hazards do not depend on one's historical process, but only on their current state (2). In line with previous literature (3,4), we relaxed this assumption by including age as a time-varying covariate. SNAC-K follows a panel data structure; as such, transition times are usually interval censored, or said to occur at some point within defined time intervals. The msm package in R was used to run the analyses, as it employs continuous time Markov models, which do not require exact transition times for events (2). However, it was possible to use the exact transition times for death, as we knew the participants exact death dates. The multi-state model for the current study included three transient states corresponding to different degrees of frailty status (robust, mild frailty, and moderate and severe frailty) and death as an absorbing state. To facilitate model convergence, and in line with recommendations concerning models involving ordered levels of diseases (2), transitions were only allowed between adjacent frailty states, and to death (**eFigure 2**). Right censoring was employed for dropouts. Initial transition intensities were selected based on reported frailty index transition probabilities in previous studies (5-7), and then the crudeinits.msm function was used to obtain plausible values in line with the data. Age (centred at 60) was used as the timescale.

Frailty state-specific life expectancies

The ELECT package in R was used to estimate state-specific life expectancies with 95% confidence intervals for robust persons at age 60, also stratified by sex and birth year (3). The fitted continuous-time multi-state survival models were used, and simulations with 1000 repetitions were implemented to assess the uncertainty (3). The maximum age that participants were allowed to live to was 107 years, as this was the maximum age at baseline in SNAC-K. State-specific life expectancies forecasted how one's remaining years of life would be distributed across states of robustness, mild frailty, and moderate and severe frailty, given they were robust at age 60. We used the fitted multi-state survival model to produce estimates for five arbitrary birth years (1900, 1910, 1920, 1930, 1940). These were estimates based on our fitted model, and not actual observed life expectancies of participants born in these years. **eFigure 3** presents these forecasted frailty state-specific life expectancies for people born in the years 1898 to 1945, and **eTable 8** contains the number of participants born in each year. State-specific life expectancies were presented as number of years as well as the proportion (number of years in a state divided by total number of years in all states) of remaining life spent in different states.

References

1. Brunson JC, Read QD. ggalluvial: Alluvial Plots in 'ggplot2.' R package version 0.12.3. [Internet] 2020 [cited 2023 Feb 7]. Available from: http://corybrunson.github.io/ggalluvial/
2. Jackson C. Package 'msm.' [Internet] 2022 [cited 2023 Feb 7]. Available from: https://cran.r-project.org/web/packages/msm/vignettes/msm-manual.pdf
3. van den Hout A, Sum Chan M, Matthews F. Estimation of life expectancies using continuous-time multi-state models. *Comput Methods Programs Biomed* 2019;178:11–8. doi: 10.1016/j.cmpb.2019.06.004
4. Vermunt L, Sikkes SAM, van den Hout A, Handels R, Bos I, van der Flier WM, et al. Duration of preclinical, prodromal, and dementia stages of Alzheimer's disease in relation to age, sex, and APOE genotype. *Alzheimers Dement*. 2019;15(7):888-98. doi: 10.1016/j.jalz.2019.04.001
5. Romero-Ortuno R, Hartley P, Knight SP, Kenny RA, O'Halloran AM. Frailty index transitions over eight years were frequent in The Irish Longitudinal Study on Ageing. *HRB Open Res* 2021;4:63. doi: 10.12688/hrbopenres.13286.1
6. Larsen RT, Turcotte LA, Westendorp R, Langberg H, Hirdes JP. Frailty Index Status of Canadian Home Care Clients Improves With Exercise Therapy and Declines in the Presence of Polypharmacy. *J Am Med Dir Assoc* 2020;21(6):766-71.e1. doi: 10.1016/j.jamda.2020.01.004
7. Ohashi M, Yoda T, Imai N, *et al*. Five-year longitudinal study of frailty prevalence and course assessed using the Kihon Checklist among community-dwelling older adults in Japan. *Sci Rep* 2021;11(1):12399. doi: 10.1038/s41598-021-91979-6
